# Precision Food Parenting: A Proposed Conceptual Model and Research Agenda

**DOI:** 10.3390/nu13103650

**Published:** 2021-10-19

**Authors:** Tom Baranowski, Debbe Thompson, Sheryl O. Hughes, Teresia M. O’Connor

**Affiliations:** USDA/ARS Children’s Nutrition Research Center, Department of Pediatrics, Baylor College of Medicine, 1100 Bates St., Houston, TX 77030, USA; dit@bcm.edu (D.T.); shughes@bcm.edu (S.O.H.); teresiao@bcm.edu (T.M.O.)

**Keywords:** family, feeding style, food parenting practices, child dietary intake

## Abstract

Precision medicine, nutrition and behavioral interventions are attempting to move beyond the specification of therapies applied to groups, since some people benefit, some do not and some are harmed by the same therapy. Instead, precision therapies are attempting to employ diverse sets of data to individualize or tailor interventions to optimize the benefits for the receiving individuals. The benefits to be achieved are mostly in the distant future, but the research needs to start now. While precision pediatric nutrition will combine diverse demographic, behavioral and biological variables to specify the optimal foods a child should eat to optimize health, precision food parenting will combine diverse parent and child psychosocial and related variables to identify the optimal parenting practices to help a specific child accept and consume the precision nutrition specified foods. This paper presents a conceptual overview and hypothetical model of factors we believe are needed to operationalize precision food parenting and a proposed research agenda to better understand the many specified relationships, how they change over the age of the child, and how to operationalize them to encourage food parenting practices most likely to be effective at promoting healthy child food choices.

## 1. Introduction

The most common causes of mortality in advanced economic countries are cardiovascular diseases (CVD) and cancers [1]. Diet substantially contributes to these illnesses [2]. Dietary preferences and practices are learned in childhood [3,4,5], thereby offering a strategically important opportunity to influence lifelong intake. Existing prescriptions for health are designed for groups of people, but there has been substantial variability in response to these group prescriptions [6]. Research and intervention to improve child health status are moving toward “precision nutrition” [7,8], i.e., the specification of nutrients, foods, or food patterns, most appropriate to promoting an individual’s health (as opposed to generic dietary prescriptions which may not be relevant for a particular child). Precision nutrition is an evolving field, but considers a person’s health/disease status, genetics, metabolomics, microbiome, current food availability (e.g., grocery store, restaurant, home), and personal characteristics (e.g., previous food consumption that day, food preferences, etc.) to inform individualized guidance regarding the optimum nutrient intake to promote health for that individual person, such as a child. This is a work in progress. Guidelines have been published for evaluating whether genotype-based dietary advice can be evaluated as convincingly, probably, possibly, or not more effective than population prescriptions; this is based on the number of published studies supporting the findings, and whether the relevant mechanism is understood [9]. How personalized nutrition interventions could best be delivered is still under investigation, but may involve web-based approaches [10] or the use of consumer technology to deliver content in real time.

To keep pace with consumer expectations and the available science and technology, behavioral nutrition must move toward precision dietary behavior change [11,12], i.e., transitioning away from generic, one-size-fits-all dietary change procedures used to influence everyone (groups of people) to procedures tailored to most likely to be effective with a specific individual and the situations or environments they encounter. How to achieve this goal, however, is unclear.

Many factors influence child dietary intake [13,14,15], the most important of which need to be taken into consideration when making personalized dietary change prescriptions. High among these influences, especially for younger children, is parents [16]. Working with families to enhance the health of the child(ren) by way of dietary change has a long history [17]. Concerns exist about the effectiveness of family-based interventions for child obesity treatment [18] and dietary changes in particular [19]. Most studies attained minimal to no effects (in BMI or diet) despite incorporating a kitchen sink of behavior change techniques [18]. Inconsistent results were also reported in a review of family-based child dietary change interventions which assessed parent involvement [19]. Thus, while family-based interventions have become common, even popular [18,20], innovations are needed in how these interventions are designed, delivered and evaluated.

Food parenting practices (FPP) (i.e., the specific behaviors parents use to influence their child’s dietary intake [21]) provide a tool for individualizing dietary change prescriptions. While much progress has been made in specifying what food-related parenting practices exist [21,22,23], the circumstances in which they are most likely to be volitionally and effectively used need to be addressed. Which parenting practices are employed in any situation, however, reflects a selection by the parent from among diverse considerations and may change over time given the results of its use at a previous time (i.e., feedback).

Precision food parenting (PFP) attempts to enhance the health of a child by encouraging the child to consume healthier foods. Since the parent is a primary influence on what their child eats, PFP would specify whatever the parent can do either immediately at a meal or prior to a meal, or at snacking opportunities (i.e., FPP), taking into account contextual factors and child characteristics to increase the likelihood of the child selecting and consuming a healthier diet. PFP could take into account anticipation/understanding of more biological influences associated with precision medicine and nutrition, and social structure influences associated with precision public health [24].

**Figure 1:**
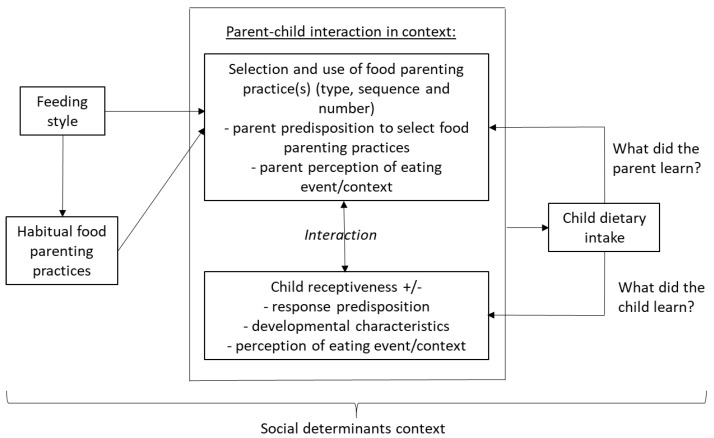
Model for precision food parenting practices interventions.

We intend this paper to be conceptual, introducing new ideas and new ways of thinking about old ideas, rather than presenting a narrative review of the literature on a single relationship. Figure 1 presents a hypothesized conceptual model intended to specify “precision” in regard to food parenting. PFP requires a highly specified model of how the FPP selections are made, to guide where and how change might be encouraged. The model organizes multiple parent behavior and child receptiveness factors influencing a child’s dietary intake, the complex interplay between and among these factors, and thereby the considerations when implementing PFP targeting children. The objective of this conceptual model and paper is to present the complexities in applying FPP to increase child healthier food intake, and to explicate research issues (Table 1) needed to enhance its future use. This model is a work in progress, not currently fully specified. Each section below explicates what is known about each construct, and what is needed to enhance PFP.

## 2. Feeding Styles

The overall parent–child relationship can be operationalized by Parenting Style, i.e., “a context that moderates the influence of specific parenting practices on the child” [25]. Parenting style is an emotion-laden construct concerning the longer-term relationship established by the way the parent treats or acts towards the child. Two constructs have been elucidated that characterize styles: the parent’s demandingness or control of the child’s behavior and their responsiveness to the child’s concerns and behaviors [26]. Crossing the two parenting style dimensions (demandingness and responsiveness) results in four categories: authoritative (high demandingness and responsiveness), authoritarian (high demandingness, low responsiveness), indulgent/permissive (low demandingness, high responsiveness) and uninvolved (low demandingness and responsiveness). Feeding style is a similar construct, but more specifically relates parenting style to a meal context [27], i.e., demandingness, structure or control in regard to eating or not eating specific foods or meals, and responsiveness to the child’s response to food acceptance and satiety. As would be expected, children of permissive and uninvolved parents had the lowest intake of nutrient rich foods [26].

Feeding style has been closely linked to child BMIz score. The indulgent feeding style has been consistently associated with higher child BMIz across multiple studies [28]. In addition, indulgent feeding style at 4–5 years of age was positively related to child BMIz at 7–9 years; and BMIz at 4–5 positively predicted indulgent feeding style and negatively predicted authoritarian feeding style at 7–9 years [27,28]. As would be expected, an authoritative parenting style involved a higher frequency of effective structure and responsive parenting practices [29]. Thus, feeding style provides an important contextual variable that should enhance understanding of the selection and use of FPP [29] (Table 1).

**Table 1:** Constructs, their relationship to precision food parenting, what is known, and priority research needs.

Construct	Relationship to PFP	What Is Known?	What Research Is Needed?
Feeding styles	The complex interplay between the parent and the child defined by two dimensions: the parents’ demaningness and responsiveness, both in regard to the child’s food behavior	Crossing the two dimensions yields four categories of relationship: authoritative, authoritarian, permissive and uninvolved [29]. Indulgent practices lead to worse outcomes (diet, BMI) [30]. Authoritarian practices lead to the lowest BMI [31].	How consistently are the categories related to FPP, especially among goal oriented FPP? Are there critical other dimensions of this relationship?
Habitual FPP	The specific behaviors that parents use without forethought with the intent to influence their child to eat specific foods, whether successful or not.	There are 17 proposed categories of FPP [23]. Some practices are likely to increase child intake of parent specified foods, and some are not [32,33].	To what extent do FPP reflect habit, and require a habit modification approach to change? Under what circumstances do FPP result in desired child intake? What are the longer term consequences of consistent or frequent use of FPP? What are the interrelationships among use of FPP?
Parent predisposition to select FPP	Diverse variables may predict a parent’s use of FPP. Among these are personality characteristics and models of behavior, e.g., model of goal directed behavior.	The model of goal directed behavior predicted use of categories of effective and ineffective FPP [34,35,36]. Little other research has addressed prediction of parent selection of FPP.	Which variable or combinations of variables predict parent use of FPP? What are the limiting factors (e.g., stress, time constraints, depression, lack of financial resources) on the predictiveness of these variables?
Selection and use of FPP	Parents must select and use specific FPP in specific contexts/situations to influence child dietary intake	Parents tend to select FPP that are easy to use, provide benefit for their child, and/or have worked in the past [37].	Under what circumstances do parents select to use specific FPP? How consistent are parents in use of joint FPP and what are the implications of consistency/inconsistency for child intake?
Parent perception of eating event/context	Parents may vary in their perception of the context of an eating event (e.g., a special or usual event) and the extent to which the event dictates specific FPP.	Little research has addressed parent perception of eating events or FPP appropriate to the perception [37].	What are the most common categories of parents’ perception of eating events? What do parents perceive as the most appropriate FPP for each category of eating event?
Child receptiveness	Some children are receptive to any/all/some FPP, and some are not	Children are not passive recipients of FPP [37,38].	Are there effective FPP to which children are universally receptive?
Child response predisposition	Children may be receptive to a FPP or not, based on appetitive, temperament, taste sensitivities and other characteristics.	A large number of factors (e.g., child social/cultural, neighborhood) influence child dietary intake [39].	How do children with different response predispositions respond to different FPP in different situations?
Developmental characteristics	Influences on FPP and child intake vary by age and age-related characteristics of the child/adolescent.	Child temperament and appetitive and food avoidant characteristics were related to BMI [40]. Some child developmental characteristics, e.g., food neophobia or picky eating were not related to child BMI [41].	More precise definitions and operationalizations of developmental characteristics are needed. Under what circumstances are child developmental characteristics related to dietary intake and BMI, at what ages?
Child perception of eating event/context	Children may vary in their perception of the context of the eating event (e.g., a special or usual event) and the extent to which the event dictates specific behavior	Little research has addressed child perception of eating events or child behaviors appropriate to the perception.	What are the most common categories of child perception of eating events? What do children perceive as the most appropriate behavior for each category of eating event?
Child dietary intake	This objective of PFP is to enable children to consume a healthier diet.	While many possible influences on child dietary intake have been proposed, and some supported, there is no consistent findings on the relation of FPP and child dietary intake [42].	Under what circumstances do FPP influence child dietary intake?
What did the parent learn?	As a result of the use of one or more FPP on a particular occasion, the parent will have learned one or more things, and this will serve to confirm or induce change in the parent predisposition to select and employ FPP.	Little research has addressed what parents learn from employing FPP or how it influences their predispositions to use FPP in the future.	How does use of FPP in interaction with their child, peers, or other sources of parenting information influence their predisposition to select, and how to employ FPP in the future?
What did the child learn?	As a result of being on the receiving end of one or more FPP, the child could be oblivious, or adapt/modify some aspect of their receptiveness, including defensive behaviors for future attempts. It is likely that different children will respond to the same FPP in different ways.	Little research has addressed what children learn from receiving FPP or how the experience results in changes in their receptiveness.	Under what circumstances, what and how does a child learn from a parent’s use of FPP, and how does this experience of related comments from peers and the media impact their future receptiveness.
Social determinants context	All parent and child behaviors and their interactions are performed in cultural and socioeconomic-demographic context.	There are inconsistent findings in regard to how context influences any of the above, perhaps due to complexity [43].	How, and under what circumstances, do any of the above outcomes, relationships or other vary by cultural and socioeconomic-demographic context?

Legend: PFP = precision food parenting; FPP = food parenting practices; BMI = body mass index.

## 3. Habitual Food Parenting Practices

Early efforts at measuring FPP included parent normative expectations, supportive, permissive, control, self preparation (low demandingness) and modeling practices [44]. Over the next two decades, measurement of FPP across studies varied with different items and statistics for deriving FPP categories, resulting in different names of the evolving FPP categories across studies. Recent efforts attempted to consolidate the assessment of FPP and expanded the range of practices considered. This systematic approach proposed 17 categories of FPP [23]. Based on basic concepts of parenting and child development [45,46], FPP has been divided into effective, i.e., likely to result in the parent desired child behavior, and ineffective, i.e., not likely to result in the parent desired child behavior, categories [33]. Measurement of FPP has used different methods, items have varied across studies, statistics for deriving categories of FPP varied, and the names for FPP categories differing across studies leading to limited clarity and confusion about what was measured.

The selection of FPP(s) in any situation likely reflects a habit, i.e., the mechanical non-conscious selection and employment of an FPP in response to learned cues [34]. The extent to which different FPP was habitual was predicted by different psychosocial variables [34]. Two of the strongest predictors of parents’ use of ineffective vegetable FPP (a composite score reflecting professionals’ judgments about which FPP are not likely to have the parent intended influences on child intake) involved habit. The strongest positive predictor was the parent’s habit of using controlling vegetable FPP and a strong negative predictor was the parent’s habit of actively involving their child in vegetable selection [35]. When the three ineffective FPP scales were predicted separately, variables in the model accounted for 26.5%, 16.7% and 44.6% of the variances in the ineffective responsive, structure and control scales, respectively. The two strongest predictors of the use of effective vegetable FPP were the habit of active child involvement in vegetable selection and the habit of positive vegetable communications [36]. Thus, habit appears to be a key construct in understanding FPP behavior and its change [11,47]. Encouraging parents to use more effective FPP may require minimizing the habit of using ineffective FPP and helping parents develop the habit of using more effective FPP. Various procedures have been proposed to minimize less healthful habits [48] and encourage more healthful habits [49].

## 4. Parent Predisposition to Select FPP

A parent’s own health, genetics, microbiome, metabolomics, and other variables will likely influence their own nutrition behavior, and thereby their role modeling to their child among other FPP (e.g., what foods and drinks they make available at home). Parents will also have experiences with their child and other children, learned from their parents, friends, TV and other key social models, from which they will have formed attitudes, perceived norms and other personal characteristics which predispose them to the selection of FPP [50].

A model of goal-directed vegetable FPP has been proposed [51] which incorporates predisposing influences on a parent’s selection of FPP in general, and effective [36] and ineffective [35,51] practices in particular. Using psychometrically validated scales [50,52] (including attitudes, norms, perceived behavioral control, anticipated emotions, habit, competence/self efficacy, relatedness, autonomy, perceived barriers, desire and intentions toward the use of vegetable FPP [50]), the final predictive model accounted for almost 48.6% of the variance in the use of a composite of three effective vegetable FPP scales, incorporating several habit variables [36]. A model predicting a composite of three ineffective vegetable FPP scales accounted for 40.5% of the variance and included as significant several habit variables, but also autonomy, attitude and descriptive norms [35]. The model of goal directed behavior thereby provides a comprehensive set of variables that may predispose parents to use specific parenting practices, and thereby can be used to influence the selection of FPP.

## 5. Selection and Use of Food Parenting Practices

Parent selection and use of FPP are highly nuanced. Parents of children with healthier, and those with less healthy, diets both intended to provide their children with healthy foods, involved their children in food preparation, and ate evening meals together as a family [53]. A primary difference was a willingness to say “no” to unhealthy foods among parents of children with healthier diets [53].

Numerous reviews have been published on interventions to change FPP in an effort to influence child eating behaviors [19], or downstream health outcomes [13,54,55]. When parents have been included in a family change intervention, little is known about which specific FPP the parents selected, how frequently the practices were implemented, whether the parents thought the practices worked, or would use them again. A recent study asked parents of 3 to 5 year old children to select two FPP for increasing child vegetable consumption from among three categories: effective responsive, control and structure FPP, and implement for a week; after which they were intensively interviewed about the experience [37]. Responsive practices were the most commonly selected. Most parents reported selecting FPP because of their perceived ease of use (e.g., fitting into their existing routines), or perceived benefit for their child. Some selected practices that were novel for them, or more likely to be effective. This suggests that parents are open to trying novel FPP, but they need to be easy to use and perceived likely to be effective [37].

A common belief among researchers is that FPP is easier to be intervened upon and changed, compared to parenting style, because the latter is likely an indicator of personality, and involves parent emotions (which are automatically elicited) in regard to the child. The selection of FPP would be expected to vary by feeding style, reflecting what parents usually or characteristically did. However, when parents were categorized by feeding style, there was no difference in the selection of the two FPP (from 14 possible selections) by feeding styles. Little variability among the parents on the two dimensions used to define feeding styles suggested the recruited participants did not represent archetypes of the four feeding styles [37]. Greater variability on the responsiveness and demandingness scales may have led to differences in the selection of FPP, especially among parents at the extremes of the distributions.

Some children will be in the care of different caregivers, e.g., mother, grandparent, daycare provider, likely some of whom will vary in their feeding style and parenting practices [39]. Conflicts will likely arise in FPP across caregivers.

### Parent Perception of Eating Event/Context

Behaviors are performed in contexts. The parent forms perceptions of the event in which the FPP will be offered, which includes the type of location (e.g., home dining table, home in front of the TV, fast food, etc.), occasion (e.g., usual meal or snack, special occasion (e.g., birthday celebration, reward for behavior, etc.), presence and expectations/desires of others present, type of food(s), etc. The parent will have in mind a personal list of behaviors which they consider appropriate to each environment/event characteristic and combination of characteristics. For example, the authoritative parent may purchase indulgent food items for their child at a fast food restaurant for a birthday, but would not ordinarily go to a fast food restaurant or, if they do visit, would purchase from only a restrictive list of healthier food options there.

## 6. Child Receptiveness

Child receptiveness to the parenting intervention (accepting/compliant or rejecting/noncompliant) will likely be a function of the child’s developmental characteristics, response predisposition and perception of the eating event.

### 6.1. Child Response Predisposition

Most research in this area considers the child a passive recipient of the parent’s influence attempt(s), reporting little more than it influenced behavior or not [56]. A large number of child characteristics influence dietary intake [39] which would likely influence their receptiveness to an FPP, e.g., picky eating, satiety response, temperament [57], sweet and bitter taste sensitivities [58], which may influence both parent’s selection and volume of practices [57]. Consistent with expectations, child acceptance of an offered vegetable was mostly positive when parents employed non-directive control practices, but roughly equally positive and negative responses when employing responsive practices and a combination of positive and neutral responses when using structure parenting practices [37]. Parents with an indulgent feeding style were most likely to encounter negative reactions. In contrast to the usual assumption of passiveness, children have been reported to play very active roles in regard to food. Children have been reported to influence what foods the mother purchases, prepares, and consumes, and even where families eat when eating away from home [38].

### 6.2. Developmental Characteristics

A child rapidly grows physically, mentally and emotionally, which requires different ways by the parent to guide, manage or control the child’s behavior [59]. Younger children are likely responsive to more immediate influences, while older children/adolescents may be characterized by complex cognitive-motivational models. Thus, parents need to scaffold their child’s eating when they are younger and provide more autonomy as they get older.

A systematic review of 18 studies among preschoolers revealed virtually all aspects of child temperament were related in expected directions (e.g., poor self regulation, high emotionality, and high soothability were related to larger BMI increases) [40]. Similarly, another review revealed appetitive (e.g., food responsiveness, enjoyment of food, emotional overeating) and food avoidant (e.g., satiety responsiveness, emotional undereating, food fussiness) characteristics were cross-sectionally and prospectively related in expected directions with BMIz [60]. Alternatively, a review of 41 studies revealed no consistent significant relationships between food neophobia or picky eating and weight status [41].

Control or regulation of child food intake and obesity vary with age [61]. A distinction has been drawn between appetite regulation, which encompasses the many biological variables that influence a child’s appetite, and appetite self regulation, which encompasses the more psychosocial variables that influence child appetite [61]. Appetite self regulation has been divided into top-down (e.g., delay of gratification) and bottom-up (e.g., disinhibited eating) influences. Top-down influences include more cognitive self control, also called regulator factors (e.g., inhibitory control), while bottom-up, or reactive, influences include more biological factors (e.g., impulse control, approach-avoidance, reward sensitivity [62]). Among child temperaments, surgency (hyperactivity) was related to speed in eating, while effortful control (a form of self control) was related to satiety responsiveness [63].

### 6.3. Child Perception of Eating Event/Context

What constructs children use to understand and cope with these FPP may also yield important results. Child perception(s) may be influenced by the physical location (e.g., home dining room, home living room, fast food restaurant), the circumstances of the behavior (e.g., regular meal, special occasion), other actors present (e.g., other children, other family, friends, etc.). The context may include consideration of alternatives foregone (e.g., eating inside at home due to rain outside precluding travel to a special event). The child’s perception of context will often be different from that of the parent, e.g., eating at the parent’s usual workday lunch venue may be a special event to a child. While parents may expect an FPP to change a child’s behavior in a desired direction, the child’s understanding of the situation may result in an opposite or unexpected effect.

## 7. Parent–Child Interaction

Child behaviors tend to evoke FPP, and parents can influence child behavior (bidirectionality) [62]. Child development characteristics have been related to FPP [39]. Parenting influences on child intake have been demonstrated to vary across prenatal, pre-weaning, post-weaning early (6–9 mos), post-weaning later (9–12 mos), and early years [64]. Parents reporting high child food fussiness reported more healthy eating environment practices [65]. Parents with children with less healthy diets attempted to disguise vegetables and healthier foods, suggesting they were responding to their child’s fussier eating [53]. The context in which an FPP is used may affect how the child perceives it and reacts. Many things could happen. The use of any particular FPP will likely reflect the parent’s consideration of perceived relevant factors, e.g., parent restricting a child’s food intake occurs primarily when they are concerned about the child’s weight [66].

Food preference (the tastes a child enjoys) appears to be the primary influence on children’s dietary intake (what a child consumes) [67]. Repeated exposures to a new food enhanced food preference, thereby increasing the likelihood of intake [64]. Parents tend to report that their rewarding (e.g., saying nice things about the food) any dietary behavior likely increases its preference [68]. Alternatively, rewarding fruit drink intake resulted in lower fruit drink preference [69]. Perhaps a ceiling effect occurred, i.e., the reward cannot have an effect on an already highly preferred food, or parents perceive rewarding to be effective, but it actually does not increase preference for the behavior that is rewarded. Complexities in the use of rewards to influence child dietary intake have been reviewed [64].

The context is most importantly influenced by the parent’s and child’s perceptions of these contextual factors which included any personal meanings imposed on them, e.g., is the non-family member present at the eating event a close friend or a stranger, is this fast food location a place both the child and parent can select food items each enjoys, etc.?

## 8. Child Dietary Intake

The food(s) the child actually consumed is defined as the child’s dietary intake, in contrast to the foods or food patterns offered/available. From a phenomenological perspective, foods are the variables of interest, but nutrients could also be the unit of interest. From the parent–child interactive perspective, nutrients would need to be converted to food equivalents to be considered in this framework.

## 9. What Did the Parent Learn?

In light of the food related interaction and what the child ate (or did not) the parent will have learned something (e.g., the child’s response to a particular FPP, the most important influences on the child, or even nothing new occurred thus no change/updating is needed in his/her preconceptions about their parent–child interaction). The parent could also learn from comments from their primary care physician, friends, etc. What is learned may require changes in one or more of the model’s variables (e.g., attitude, self efficacy, motivation), usually called feedback.

Parents may learn different things from a similar event. Some parents may have children who ate the vegetable and saw their child expressing excitement, happiness, ownership (wanting to do the FPP themselves) and enjoyment from tasting and eating the vegetable. Other parents will have changed their strategy over the week (e.g., different vegetable served, prepared or served in a different way, or in a different location) when the child tired of the FPP [37]. It is not clear whether, when or how what a parent learns updates their attitudes, norms, etc. in regard to selecting an FPP, or the extent to which it does at all.

## 10. What Did the Child Learn?

Since children and adolescents will be at different levels of development, what they can learn will be related to their developmental stage. Different models have been proposed to represent cognitive functioning at each level [70]. Except in the simplest models of learning, the children or adolescents will be needed to report on what they understood and learned. Children will not likely be able to accurately report on what they learned until perhaps at nine or ten years of age [71,72].

## 11. Social Determinants

Numerous sociodemographic characteristics (e.g., race, ethnicity, marital status, employment status) provide context and thereby emphasize FPPs that are acceptable or prohibit/inhibit certain others. One of the most prominent of these is socioeconomic status (SES). SES is a generic category specifying a family’s position in social and economic hierarchies in which they engage, and may explain some differences in which practices parents use and how. A composite scale of SES (family income, occupation, employment status, educational attainment, and health insurance) was related to overall mortality, CVD mortality and CVD in both the US and UK; and to some extent, lifestyle mediated each outcome [73]. Use of all FPP in regard to three beverage intake variables (plain water, soft drinks and fruit juices) varied by SES [74]. SES can affect what foods are available in the home by several factors, including food insecurity [75], and thus what a parent can offer, i.e., food availability/accessibility [76]. Thus, SES is a key variable in understanding differences in parent–child interactions in regard to dietary intake.

## 12. Limitations

The research necessary to finalize a final model will take many years. Building software that simulates all the variables and empirical links, and integrates PFP with the child specific foods from precision nutrition will be expensive. The research conducted to get to those endpoints, however, should be very enlightening. While implementing the resulting software may take ample time to input the child specific variables, and the costs cannot be anticipated, the benefits to the child, and perhaps to the parents, should be substantial.

## 13. Conclusions

Since most food parenting interventions targeting child dietary change through group instruction have had minimal to no effects, new intervention approaches should consider a more comprehensive list of interacting factors. PFP practices will require an increased understanding of how the variables of each unit in Figure 1 relate to neighboring units (those with arrows), variability in response and how best to manage these relationships to promote optimal child nutrition-related health and well-being. Ideally, simulation models will be built that enable interventionists to anticipate the effects of their possible design choices, based on reasonable assumptions from the literature. To prepare the way, the research suggested in Table 1 may facilitate this and further elucidate our understanding of how these and possibly other variables interact and ultimately influence the selection and use of FPP for healthier child food-related behaviors. What exciting possibilities!
